# Seroepidemiological study of rubella in Vojvodina, Serbia: 24 years after the introduction of the MMR vaccine in the national immunization programme

**DOI:** 10.1371/journal.pone.0227413

**Published:** 2020-01-13

**Authors:** Aleksandra Patić, Mirjana Štrbac, Vladimir Petrović, Vesna Milošević, Mioljub Ristić, Ivana Hrnjaković Cvjetković, Snežana Medić

**Affiliations:** 1 Faculty of Medicine, University of Novi Sad, Novi Sad, Serbia; 2 Centre for Virology, Institute of Public Health of Vojvodina, Novi Sad, Serbia; 3 Centre for Disease Control and Prevention, Institute of Public Health of Vojvodina, Novi Sad, Serbia; The Chinese University of Hong Kong, HONG KONG

## Abstract

Although rubella is usually a mild childhood disease, this infection in early pregnancy poses a serious problem due to its teratogenic effect. The goal of interrupted circulation and elimination of rubella virus was achieved in many countries in the world. The aim of this study was to determine the status of rubella immunity in Vojvodina and evaluate Serbia’s progress toward this goal. A total of 3404 residual serum samples from patients of all ages (1 to 84 years) were included in the study. Samples were collected between May 2015 and December 2017 in Vojvodina. Rubella IgG antibodies were determined using an indirect chemiluminescent immunoassay. Percentage of participants seropositive for rubella antibodies was 92.9% in the entire sample. The highest number of seronegatives was in the youngest (1 year) age group (44.7%), followed by the group aged 24–49 (6.4%) and 2–11 years (6.2%). The absence of a higher percentage of children with protective anti-rubella antibodies in the group aged 2–11 can be explained by a lower immunization coverage during certain years. Participants in the group aged 24–49 were born during the pre-vaccination period with lower rubella incidence, leading to the conclusion that not all individuals of that age came into a contact with the virus. Comparing levels of anti-rubella IgG antibodies of seropositive males and females of different ages reveals that the immunity after a contact with the virus and a previously acquired infection is stronger than the immunity after the vaccination. Although the incidence rate of rubella in Vojvodina has been low for the last ten years, there is still a risk of an outbreak due to a decrease in immunization coverage. This study shows that the percentage of susceptible individuals is high, especially considering women aged 24–49, and that additional ("catch-up") immunization is required.

## Introduction

Rubella is usually a mild childhood rash disease with rare complications. However, an infection in early pregnancy is of major public health importance due to the teratogenic effect of the virus. Primary infections during pregnancy may lead to miscarriage, fetal death, or a birth of an infant with congenital rubella syndrome (CRS). Infants with CRS may have sensorineural hearing loss, intellectual disability, heart defects, or ocular abnormalities. To address this issue, immunization against this disease has been introduced and control of CRS implemented [1–3].

The Global Vaccine Action Plan for the Decade of Vaccines (2011–2020), which was endorsed by the World Health Assembly in May 2012, has set an ambitious goal to achieve rubella elimination in at least five of the six WHO regions by 2020. The Region of the Americas has already eliminated rubella and CRS, as verified in 2015. The European Region and Western Pacific Region have set their own rubella and CRS elimination goals, while the South-East Asia Region has a target to control rubella and CRS [4]. A sustained vaccination coverage of 95% for at least one dose of the rubella-containing vaccine is recommended by the WHO to be implemented at all levels to interrupt the rubella circulation and achieve its elimination [5].

Rubella has been a mandatory notifiable disease in Serbia since 1976. Compulsory immunization of children against this disease became a part of the national immunization programme in 1993, and the MMR vaccine has been given in two doses since 1996. Immunization begins at 12 months, while the second dose of the vaccine was initially given at the age of 12. However, in 2006, the second dose was shifted to the age of seven, and the immunization at the age of 12 was continued only in children who had not received two doses of the MMR vaccine until then [6,7].

The Programme of Health Protection of Citizens against Infectious Diseases determines the rubella prevention strategy in the Republic of Serbia. The goals of the programme are: maintaining a high level of coverage (>95%) with two doses of the MMR vaccine, supplemental immunization of the rubella-sensitive population, and the establishment and maintenance of the surveillance system with the aim of reducing the rate of congenital rubella syndrome to 0.01 per 1000 of live-born children [8,9].

### Aim of the Study

The study was conducted to determine the status of rubella immunity in the population of the Autonomous Province of Vojvodina in order to identify susceptible groups in the population.

## Materials and methods

### Study area and survey design

Vojvodina is the northern province of Serbia. The population of Vojvodina was 1,931,809 in 2011, accounting for 26.9% of the population of Serbia, excluding Kosovo [10]. Its borders are with Hungary to the north, Romania to the east, Croatia to the west, and Bosnia and Herzegovina to the southwest.

Notifications of the rubella disease are delivered to the Institute of Public Health of Vojvodina. The incidence rate of the disease was presented as the ratio of the number of patients and the whole population according to the census as the denominator per 100,000 inhabitants. The vaccine coverage of the MMR vaccine (the average value of the MMR1 and MMR2 coverage, as well as the individual coverage of MMR1 i MMR2) was calculated using the administrative method from 1993 to 2017 [11]. In our study we used data obtained from the official monthly and annual reports of the Institute of Public Health of Vojvodina in the period from 1978 to 2017. Data of incidence of rubella infection were presented through descriptive epidemiological study and analysed chronologically and demographically for the observed period (from 1978 to 2017).

Analysis of disease occurrence was performed during three separate periods. The first period is the endemoepidemic (sporadic combined with outbreak cases) period between 1978 and 1993. This is the pre-immunization period. The second one is the period of localized epidemics between 1993 and 2006. The vaccine against rubella was introduced in Serbia during this period, while the rubella infection occurred in the form of localized outbreaks affecting people in collectives. The third period is the period of sporadic cases, which began after 2006.

A total of 3404 residual serum samples from patients of all ages were included in the study. Samples were collected according to the specifications of the European Sero-Epidemiology Network 2 (ESEN2) study protocol between May 2015 and December 2017 in Vojvodina. The age stratification of the sera was according to the specifications of ESEN2. Accordingly, ~100 samples were collected for each year band in the age group 0–19 years and ~200 samples for each of the age groups ≥20 years (20–24, 25–29, 30–34, 35–39, 40–49, 50–59, and ≥60), with about an equal number of samples by gender (sampling method has been described previously by Medić, et al. [12]).

For further analysis, the samples were divided into five age groups (1 year, 2–11 years, 12–23 years, 24–49 years, and 50+ years). The age distribution was adjusted according to the periods of time, used for analyses of the disease occurrence. The age distribution was created after the samples were collected and results of the serum analyses were obtained. Available information for each patient included the following: sex, age, area of residence in Vojvodina, and sample collection date. Written informed consent of study participants, or their parents or legal guardians if they were <15 years of age, was obtained.

### Determination of IgG antibodies for rubella virus

Serum samples were analysed in the Centre for Virology of the Institute of Public Health of Vojvodina, where the serological diagnostics were performed. The ADVIA Centaur Rubella IgG assay (Siemens Healthcare Diagnostics) was used for detection of rubella IgG antibodies. This immunoassay uses chemiluminometric technology. A direct relationship exists between the amount of rubella IgG activity present in the patient sample and the amount of relative light units (RLUs) detected by the system. Testing was performed on Siemens ADVIA Centaur automated analyser. As recommended by the manufacturer, all samples with anti-rubella IgG titres below 5.0 IU/mL were considered negative (susceptible to rubella). Values greater than or equal to 5.0 IU/mL, and less than or equal to 9.9 IU/mL, were considered equivocal (unknown rubella immunity status), while samples greater than or equal to 10.0 IU/mL were considered positive for IgG antibodies to rubella virus (protected against rubella). These values are based on the test manufacturer’s recommendations (Siemens Healthcare Diagnostics, Tarrytown, NY, USA) [13].

### Statistical analysis

Statistical analysis of the results was performed using the Statistical Software Package for the Social Sciences—SPSS 21. Attributive features are shown as frequencies, percentages, and 95% confidence intervals. In order to investigate the connection of features, or to generate adequate statistical models, a univariate regression analysis was used. Binary Logistic Regression Analysis was used to generate odds ratios and confidence intervals. The testing of differences in the frequency of attribute characteristics was performed using the X^2^ test. A two-factor ANOVA analysis was used to simultaneously test the effect of each independent variable on the dependent variable, also identifying a potential impact of their interaction. The Bonferroni post-hoc test was used for correction for multiple comparisons. Values with a significance level of p <0.05 were considered statistically significant.

### Ethical consideration

The study was approved by the Medical Ethics Committee of the Institute of Public Health of Vojvodina, on 14th May 2015 under the number 01-79/7a, as a part of a wider serosurvey on vaccine-preventable diseases in Vojvodina.

## Results

During the first period (pre-immunization period) in Vojvodina, rubella occurred endemoepidemically with a cyclic increase in incidence, reaching the values of over 1,000 per 100,000 inhabitants in epidemic years. The highest incidence rate of 1,402.1 per 100,000 inhabitants was reported in 1989.

The second period is marked by the introduction of the vaccine against rubella, while the rubella infection occurred in epidemic form, and the disease was registered with a declining trend until 2005. The highest incidence rate of 939.1 and 653.6 per 100,000 inhabitants was reported in 1995 and 1994, respectively, while the lowest coverage of the MMR vaccine (82%) was registered in 2000.

**Fig 1:**
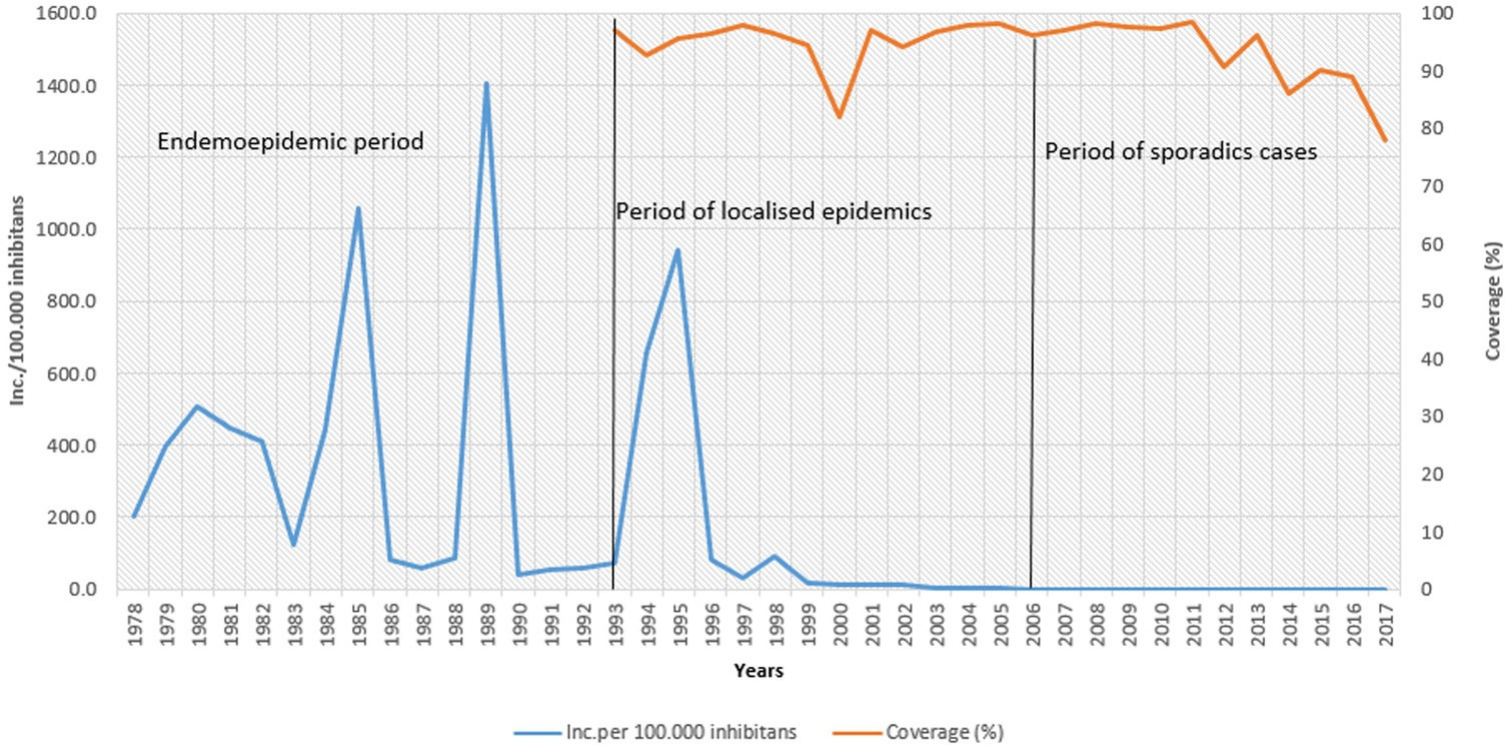
The incidence of rubella in Vojvodina between 1978 and 2017, and the average of MMR1 and MMR2 vaccination coverage between 1993 and 2017.

The third period began in 2006, when no cases were reported for the first time, while only sporadic cases per year were registered in 2009, 2012, and 2015. Since 2012, there has been a declining trend in the MMR vaccine coverage in the territory of Vojvodina, with the lowest coverage registered in 2017 (78%). (Fig 1, S1 Table).

In this study of population immunity against rubella virus in Vojvodina, 3404 serum samples were analysed. The age of examined patients ranged from 1 to 84 years. Among participants included in the study, there were 1652 females and 1752 males. Immunocompromised individuals and recent recipients of blood and blood products were excluded. The percentage of participants seropositive for rubella antibodies was 92.9% (95% Cl, 92.0–93.8%) in the entire sample. There were 194 individuals (5.7%) without antibodies against rubella, while 1.4% of the participants had borderline values.

The values of rubella seropositivity for males and females were 92.5% and 93.3%, respectively. Although a higher proportion of seropositivity was found in females compared to males, the difference was not statistically significant (p = 0.291).

The highest number of seronegativity was found in the youngest (1 year) participant group (44.7%), followed by the age group of 24–49 years (6.4%) and the group of children aged 2–11 years (6.2%). Although the participants in the age group of 24–49 years were born during the pre-vaccination period, they have a slightly higher seronegativity (6.4%) compared to the group of ≥50 years. This difference is statistically significant (x^2^ = 20.973; p <0.001). The percentage of equivocal results in both females and males in the age groups of 1 year and 12–23 years ranged between 2.2 and 4.0%, while the percentage in the other age groups ranged between 0.4% and 1.2% (Table 1).

**Table 1:** Seroprevalence of anti-rubella antibodies according to the age and gender in Vojvodina, Serbia, 2015–2017.

		Seropositive		Seronegative		Equivocal		Total		
Age group		No	% (95%CI)	No	% (95%CI)	No	% (95%CI)	No	OR (95%CI)	p
Males	1y	44	48.4 (38.1–58.7)	45	49.5 (39.2–59.8)	2	2.2 (0.8–5.2)	91	250.0 (31.0–1717.3)	<0.001
	2-11y	438	93.8 (91.6–95.9)	25	5.4 (3.4–7.4)	4	0.9 (0.0–1.8)	467	13.3 (1.8–99.1)	0.011
	12-23y	484	95.8 (94.1–97.6)	8	1.6 (0.5–2.7)	13	2.6 (1.2–4.0)	505	3.8 (0.5–30.5)	0.210
	24-49y	420	92.9 (90.5–95.3)	28	6.2 (4.0–8.4)	4	0.9 (0.0–1.8)	452	15.6 (2.1–115.3)	0.007
	≥50y	235	99.2 (98.1–100.3)	1	0.4 (-0.4–1.2)	1	0.4 (-0.4–1.2)	237	1.0*	
	Total	1,621	92.5 (91.3–93.7)	107	6.1 (5.0–7.2)	24	1.4 (0.8–2.0)	1,752		
Females	1y	30	60 (46.4–73.6)	18	36 (22.7–49.3)	2	4.0 (1.4–9.4)	50	33.3 (10.7–105.6)	<0.001
	2-11y	361	91.9 (89.2–94.6)	28	7.1 (4.6–9.6)	4	1.0 (0.0–2.0)	393	4.6 (1.6–13.2)	0.005
	12-23y	499	96.3 (94.67–97.9)	7	1.4 (0.4–2.4)	12	2.3 (1.0–3.6)	518	0.8 (0.2–2.8)	0.751
	24-49y	416	92.9 (90.5–95.3)	30	6.7 (4.4–9.0)	2	0.4 (0.2–1.0)	448	142.9 (1.5–12.3)	0.007
	≥50y	236	97.1 (94.9–99,2)	4	1.6 (0.0–3.2)	3	1.2 (0.2–2.6)	243	1.0*	
	Total	1,542	93.3 (92.1–94.5)	87	5.3 (4.2–6.4)	23	1.4 (0.8–2.0)	1,652		
Total	1y	74	52.5 (44.3–60.7)	63	44.7 (36.5–52.9)	4	2.8 (0.1–5.5)	141	76.7 (29.9–196.7)	<0.001
	2-11y	799	92.9 (91.2–94.6)	53	6.2 (4.6–7.8)	8	0.9 (0.3–1.5)	860	6.2 (2.5–15.7)	<0.001
	12-23y	983	96.1 (94.9–97.3)	15	1.5 (0.8–2.2)	25	2.4 (1.5–3.3)	1,023	1.4 (0.5–3.9)	0.505
	24-49y	836	92.9 (91.2–94.6)	58	6.4 (4.8–8.0)	6	0.7 (0.2–1.2)	900	6.5 (2.6–16.4)	<0.001
	≥50y	471	98.1 (96.9–99.3)	5	1.0 (0.1–1.9)	4	0.8 (0.0–1.6)	480	1.0*	
	Total	3,163	92.9 (92.0–93.8)	194	5.7 (4.9–6.5)	47	1.4 (1.0–1.8)	3,404		

*Reference value

OR–Odds Ratio; CI—Confidence Interval; p–probability

Binary Logistic Regression Analysis was used to generate odds ratios and confidence intervals.

In the entire sample, using the age group of ≥50 years as a comparator, the prevalence of rubella seronegative results was significantly higher in the following age groups: 1 year, 2–11 years, and adults aged 24–49 years (OR 76.73, OR 6.24, and OR 6.54, respectively). Similar results are obtained in the same age groups for males and females. The results are shown in Table 1.

Analysing the percentage of seropositive subjects in the three observed periods, we found that there were statistically significantly fewer seropositives in the age group of <11 years compared to the other two observed age groups (x^2^ = 71,910; p <0.001) (Table 2).

**Table 2:** Percentage of seropositive and seronegative according to the age groups.

		Age group								p
		≤11		12–23		24+		Total		
		N	%	N	%	N	%	N	%	
Rubella CLIA IgG results	Seronegative	128	12,8%	40	3,9%	73	5,3%	241	7,1%	
	Seropositive	873	87,2%	983	96,1%	1.307	94,7%	3.163	92,9%	<0.001
	Total	1.001	100,0%	1.023	100,0%	1.380	100,0%	3.404	100,0%	

p–probability; X^2^ test was used to determine whether there is a statistically significant difference in the percentage of seropositives during the three observed periods.

Comparing the levels of anti-rubella IgG antibodies across these three periods, we found that in the period of single cases of the rubella disease, the lowest levels of antibodies were found in the 1-year old participants compared to the age group of 2–11 years old. The highest levels of IgG antibodies were detected in the age group older than 24 years, which belongs to the period of massive epidemics of the rubella infection.

**Fig 2:**
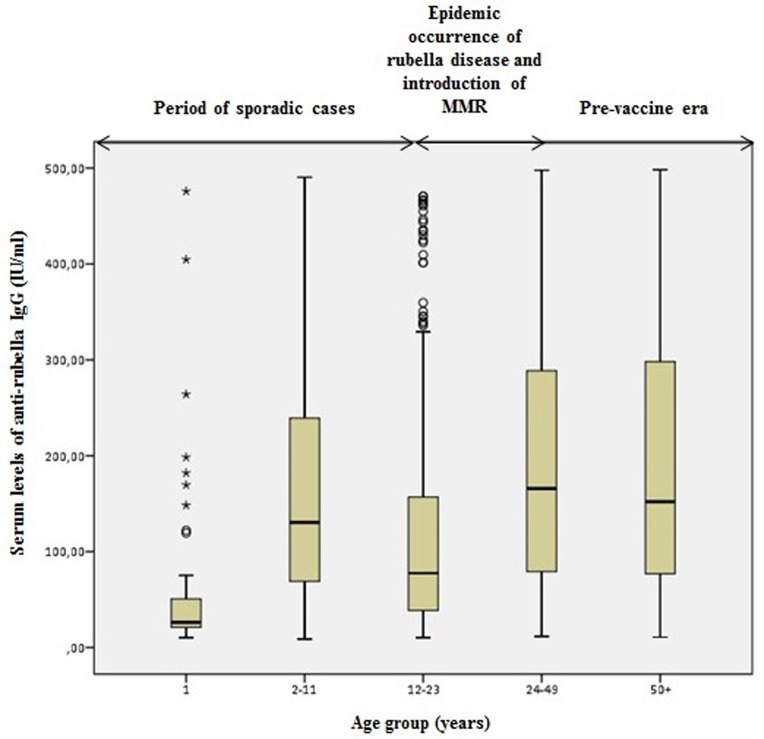
Serum level of anti-rubella IgG in seropositive females.

**Fig 3:**
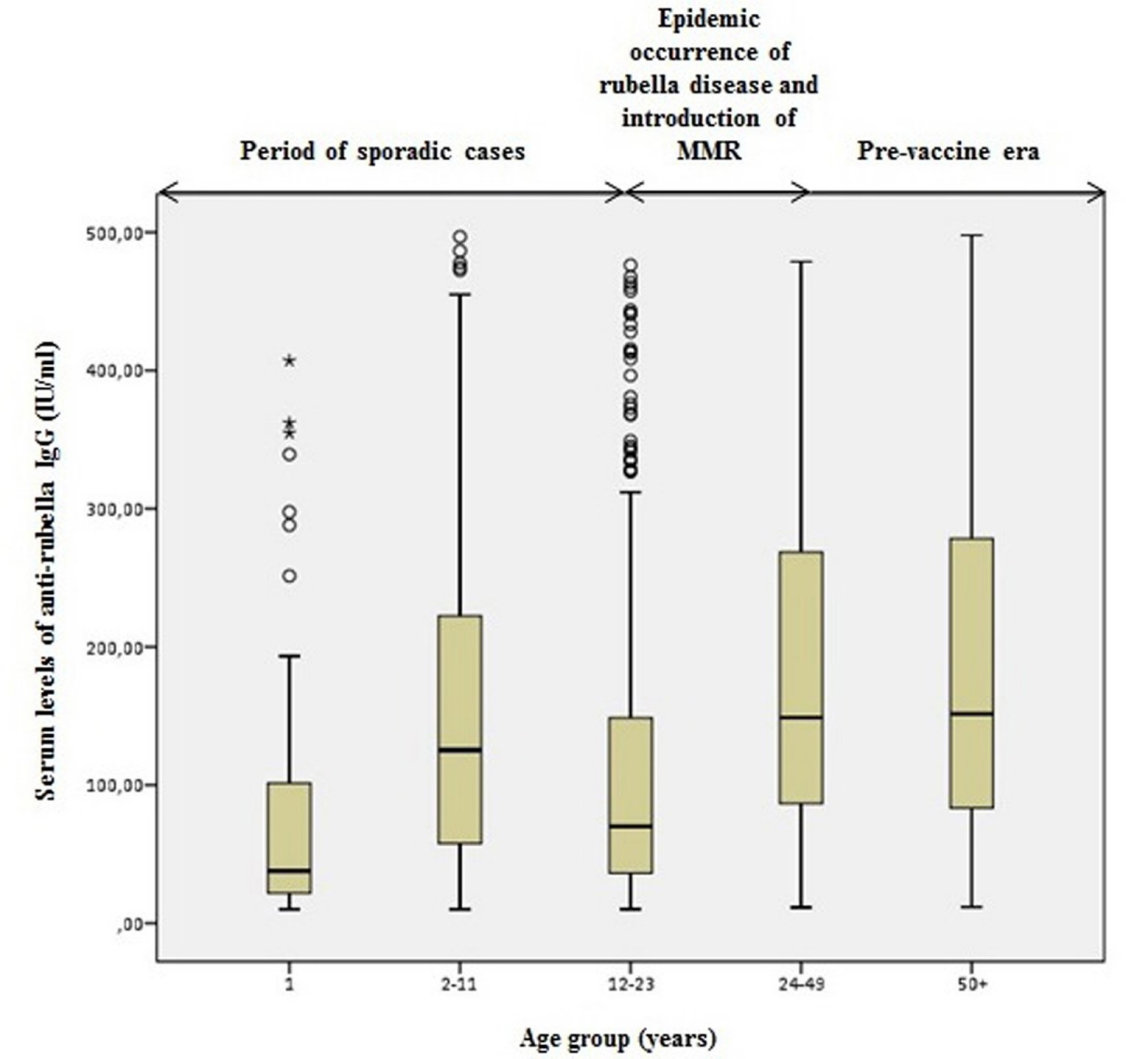
Serum level of anti-rubella IgG in seropositive males.

In the age group of 12–23 years, which belongs to the period of localized epidemics of the rubella infection and the introduction of the MMR vaccine in the population of Vojvodina, the levels of antibodies were low for both males and females compared to the other age groups (Fig 2 and Fig 3).

Also, we analysed whether there was a statistically significant difference in measured antibody levels in different age groups (during the three observed periods) in seropositive subjects. The analysis showed that there was a statistically significant difference in antibody levels compared to the observed age groups (ANOVA; F = 99,394; p <0.001). The Bonferroni post hoc test showed that patients aged 12–23 years had statistically significantly lower antibody levels compared to those aged <11 years (p <0.001), as well as those aged 24+ years (p <0.001). Patients older than 24 years (there is no difference between the age group of 24–49 years and those older than 50) also had statistically significantly higher antibody levels compared to those younger than 11 years (p<0.001). Patients from the oldest age group had statistically significantly higher antibody levels than subjects aged 12–23 years (p <0.001) (Table 3, S1 Fig).

**Table 3:** Antibody levels in different age groups (during the three observed periods) in seropositive participants.

	N	Mean	Std. Deviation	p
1–11	711	152,9883	118,66190	
12–23	926	108,1632	102,87936	<0.001
24+	890	185,4685	129,72264	
Total	2.527*	148,0019	121,81713	

* Samples that had antibody level values greater than 500 IU/ml were not included in this analysis, since we did not have an accurate antibody level value for them. The assay we used can determine antibody level values only up to 500 IU/ml.

The two-way ANOVA analysis and the Bonferroni post hoc test were used to determine whether there is a statistically significant difference in the antibody levels in seropositive participants during the three observed periods.

We further tested the effect of gender and age groups on antibody levels, as shown in the Table 4. Using the Two-way ANOVA analysis, we tested the individual effect of gender and age groups, as well as a potential impact of their interaction, on antibody levels. The effect of gender and age group interaction on antibody levels was not statistically significant (F = 0.359; p = 0.698). There was a statistically significant individual effect of age groups (subjects’ age) on antibody levels (F = 99,472; p<0.001), but no individual effect of subjects’ gender (F = 0.981; p = 0.322).

**Table 4:** Influence of gender and age on serum level of anti-rubella IgG in seropositive participants.

gender	Age group (years)	Mean	Std. Deviation	N
male	1–11	148,2071	118,01142	388
	12–23	107,4753	104,88812	454
	24+	184,4087	127,10503	445
	Total	146,3559	121,16096	1.287
female	1–11	158,7317	119,36821	323
	12–23	108,8248	101,01651	472
	24+	186,5283	132,42313	445
	Total	149,7103	122,51995	1.240
Total	1–11	152,9883	118,66190	711
	12–23	108,1632	102,87936	926
	24+	185,4685	129,72264	890
	Total	148,0019	121,81713	2.527

The two-way ANOVA analysis was used to determine whether there is a statistically significant influence of gender and age on antibody levels in seropositive participants during the three observed periods.

## Discussion

According to the latest reports, epidemiological situation of rubella in most EU countries is favourable. In 2017, 11 European countries reported 696 rubella cases, which represented a marked decrease from 1,264 and 2,161 cases reported in 2016 and 2015, respectively. During 2017, in the territory of 17 European countries there were no rubella cases. The highest number of cases was reported in Poland (496, where the last epidemic was registered), Germany (73), Italy (67), and Austria (39) [4]. In 2016, six cases of CRS were reported in 42 European countries which keep records and report this syndrome [14].

During the ten-year period (2008–2017), only three cases of rubella infection were reported (in 2009, in 2012, and in 2015). The reservoir of infection in all three cases was not determined. Therefore, the actual number of cases might have been higher [6,7].

In this study, the percentage of participants seropositive for rubella antibodies was 92.9% in the entire sample. This percentage of seropositivity is similar to the percentage obtained in a study in Luxembourg in 2004 [15], but lower than the percentage obtained in Hungary, Slovenia, and Slovakia, and higher than the one found in Romania and Bulgaria in 2008 [16]. A higher percentage of seropositives in Hungary, Slovenia, and Slovakia can be explained by a >95% coverage of the MMR vaccine in these countries, but also by a previously acquired immunity of the population following a natural infection. [17].

In contrast to our study, in the study conducted in Vojvodina between 1978 and 1984 on a sample of 1,550 individuals aged from six months to 60 years, the percentage of susceptible individuals aged ≥20 years ranged from 0% to 15% [18]. The period of research in this study belongs to the pre-vaccination era. In our study, the percentage of susceptible individuals in the group aged ≥20 years was 4.2% (6.4% in the age group of 24–49 years, and 1% in the age group of ≥50 years). These data only confirm that the introduction of the MMR vaccine has significantly reduced the number of susceptible individuals in the Vojvodina population. Despite the high percentage of people with protective anti-rubella IgG antibodies, the number of susceptible individuals is still higher than recommended (<5%) by the World Health Organization. The goal of rubella and CRS elimination can only be achieved when the population susceptibility is below the level that can sustain transmission. Thus, a population immunity of at least 95% is recommended to interrupt the transmission and eliminate rubella. Susceptibility should be lower than 5% especially in children and women of generative age [5,19].

In our study, the highest number of seronegatives is found in the youngest (1 year) age group, followed by the age group of 24–49 years, and the group of children aged 2–11 years.

A lower percentage of seropositive participants in the group of children aged 1 year (52.5%) can be explained by the fact that a portion of the subjects in this group have lost protective maternal antibodies but have not yet received the first dose of the MMR vaccine. The level of antibodies transferred from mother to child shows an exponentially declining trend, resulting in the loss of protection against rubella after a few months–well before the age of the first MMR vaccine dose [20,21]. The first dose of the MMR vaccine, which should be administered between 12 and 15 months of age, is often received later and, therefore, children aged 1 year have no protective antibodies. A similar result was obtained in a study conducted in Turkey, where the percentage of seropositives in the group of children aged 1–2 years was 51.9% [22]. In a study conducted in Germany on a representative sample of children under 17, it was determined that the percentage of seropositives in children aged 1–2 years was 69.3% [23].

In our study, the percentage of seropositives in children aged 2–11 years was 92.9%. The absence of a higher percentage of children with protective anti-rubella antibodies in this group can be explained by a lower immunization coverage of the MMR vaccine during certain years in the period from 2006 to 2017. Immunization coverage on the territory of Vojvodina was lower than 95% in 2012 (91%), 2014 (86%), 2015 (90%), 2016 (89%), and 2017 (78%). Decreasing immunization trend in Vojvodina can be explained by a reduction in timely availability of the MMR vaccine in Serbia from 2012–2016, as well as an increased activity and media presence of the anti-vaccination movement in the Balkan countries, which has contributed to a surge in vaccine scepticism and vaccine hesitancy [6,24,25]. Maintaining such a negative trend in immunization coverage leads to an increase in the number of susceptible populations. This creates conditions for the importation and spread of the disease which was previously successfully prevented by immunization. Due to this situation, there was a measles epidemic outbreak in Vojvodina from November 12, 2017 to June 30, 2018, during which 177 cases were reported [24]. There has been no increase in notification of rubella cases in Vojvodina. That happened probably because, if one dose of the MMR vaccine was higher in coverage than the other in a given year, there is a possibility that a certain level of antibodies was created, because one of MMR doses had higher coverage and there is a likelihood that more children received at least one dose of the vaccine. This means that, although there may be a lower level of antibodies in certain age groups, it seems to be sufficient to prevent the outbreak of rubella virus epidemics in the population, which was not the case with measles. In a study conducted in the northern part of Greece, the percentage of seropositives in children aged 6–10 years (71.8%) and 11–15 years (77.1%) was significantly lower than the percentage obtained in our study [26]. A lower percentage of seropositives was also found in a study in Germany, with the result of 82.2% in children aged 3–6 years, and 80.6% in children aged 7–10 years [23].

Although the participants in the age group of 24–49 years were born during the pre-vaccination period, they have a slightly higher seronegativity (6.4%) compared to the group of ≥50 years. This difference is statistically significant (x^2^ = 20.973; p <0.001). A possible explanation can be found in the registered incidence of less than 100 per 100,000 inhabitants in Vojvodina in certain years during the period after 1986, which leads to the conclusion that not all individuals of that age came into a contact with the virus. This finding underlines the need for a vaccination strategy targeting older people as well, especially women of generative age [19]. Our results are quite different from those obtained in a study carried out in Italy, where the percentage of seropositive women of generative age (18–49 years) was only 82.3% [27]. Much better results were obtained in a study in the Netherlands [28] and in part of Spain—Catalonia [29], which found >95% of seropositive women of generative age.

A low number of seronegatives was also found among the participants aged 12–23 years (1.5%). Individuals in this age group were born between 1993 and 2005. The introduction of the second dose of the MMR vaccine into Serbia’s immunization schedule in 1996 increased the likelihood of children receiving at least one dose of the vaccine and thus acquiring vaccine immunity. Also, the introduction of the anti-rubella vaccine was suppressed by an intense rubella infection in the population during the 1994–1995 epidemics, when more than 32,000 people were infected. Therefore, in this period, it was possible to obtain immunity from a rubella infection. A higher percentage in the age group of 16–20 years (8.7%) was obtained in a study conducted in the northern part of Greece [26].

The lowest number of seronegative participants was found in the group aged 50+ (1.0%). People from this age group were born before routine vaccination against rubella. In the pre-vaccination era, the average annual incidence of rubella was high. A large number of people could have come into a contact with the rubella virus as early as childhood and more frequently in older age, and therefore, were more likely to have acquired immunity from a natural infection.

Comparing levels of anti-rubella IgG antibodies of seropositive males and females of different ages reveals that those born before 1993 generally show statistically significantly higher levels of rubella-specific IgG antibodies compared to those born during the period when the MMR vaccination was introduced. This is consistent with the fact that the immunity after a contact with the rubella virus and a previously acquired infection is stronger than the immunity which develops after the vaccination. These results are consistent with the results obtained by Smits G. et al, in the Netherlands study [28].

Individuals born between 1993 and 2006 (aged 12–23 years) have statistically significantly lower levels of anti-rubella IgG antibodies. During this period, the MMR vaccine immunization programme was introduced and the vaccination schedule changed. Introduction of the vaccine against rubella limited the circulation of the virus in the population. However, the coverage of the vaccination in this period was lower than required, so it is possible that many individuals did not receive both doses of the MMR vaccine. Consequently, the anti-rubella IgG antibody levels ​​are lower. This is confirmed by the study by Pebody RG. et al, who highlighted that the geometric mean antibody concentration of rubella was significantly higher in children who have received a second dose of the MMR compared to those who have received a single dose [30]. Also, in this age group there was a higher percentage of equivocal results. It is very likely that vaccine immunity has declined among these individuals because they have not received both doses of the vaccine. Consequently, it is not only individuals with equivocal test results who present a problem in this age group. After some time, the problem may also be with individuals who currently have sufficient levels of protective antibodies, but–due to the possible administration of only one dose of the MMR vaccine–might experience a more rapid decrease in antibody levels.

In children born after 2006, the lowest antibody level was among those aged 1 year. Most children in that age group have lost maternal antibodies, so their existing antibodies are the result of a single dose of the MMR vaccine. Although children are expected to develop normal antibody titres in response to the initial MMR dose, some children develop low antibody levels to all three antigens in the MMR vaccine, and some (the non-responders) develop no antibodies. Low antibody levels may be caused by a small proportion of complete vaccination failures, impotent vaccine, incorrect delivery, or individual factors related to an underdeveloped immune system [30,31]. In the group of children aged 2–11 years, the level of anti-rubella IgG antibodies was higher since many of them have received both doses of the MMR vaccine.

Our study might have some limitations. First, for the duration of the study, the number of samples of the youngest participants was slightly lower than planned. The study used serum samples of patients from routine work, from which a serum bank was made to test the immunity of the population to certain pathogens (measles, varicella, hepatitis A, hepatitis B and rubella). Due to the analyses done for all these agents, we had 26% less serum in the age group of 1 year for the purposes of our research on the immunity to rubella virus. Regarding the OR result for the 1y group, we understand that the OR number is high, but we can try to explain. The >50y age group was taken as a reference value for comparisons. This age group belongs to the unvaccinated population which has witnessed high incidence rates of rubella during major epidemics. Of the 237 subjects over the age of 50, only one male subject was seronegative, and one male subject had an equivocal finding. As many as 99.2% were seropositive, so the OR for male subjects aged 1 was 250, and its confidence intervals were wide, which could potentially limit the ability for adequate interpretation of the results. Second, equivocal results were not retested in terms of a definitive laboratory classification (positive or negative). Third, standardization was not performed due to a lack of a reference (comparative) panel. For epidemiological purposes, the enzyme immunoassay (EIA) is a widely used method to detect antibodies to rubella in serum. However, variations in both sensitivity and specificity of several commercial EIAs have been reported, as well as inter-laboratory variability when the same assay is used. Therefore, the European Sero-Epidemiology Network recommends standardization of assays to ensure direct comparability of seroepidemiology results obtained during different projects. Fourth, we did not collect individual immunization coverage of participants, migrant status, education level and other personal data. Therefore, we could only guess whether specific anti-rubella IgG antibodies originated from vaccination or natural infection. However, that was not the initial idea of the study. This was the first study on the immunity of the Vojvodina population to rubella virus. Some future research could provide additional data. Fifth, it is quite possible that the population-level immunity to rubella virus may be lower than the results obtained in this study, since immunocompromised individuals were excluded. For that reason, we think that further research is needed in the future, which we are planning to do. The collected serums were from Vojvodina residents who came to our institution for laboratory testing during the study period. Subjects' participation in the study was voluntary. The study included the subjects who gave their written informed consent to participate in the study. It is possible that some refused to participate because they avoided vaccination (either for themselves or for their children), but we do not currently have information about it.

## Conclusion

Seroepidemiological study in the population of Vojvodina shows that the percentage of susceptible individuals is higher than 5%, which leads us to suspect that there is an increased risk of the rubella disease, which is especially dangerous in women aged 24–49. Although the incidence rate of rubella in Vojvodina has been continuously below 1 per 100,000 for the last ten years, due to the percent of seronegative individuals in our investigation as well as obvious decrease in the coverage of the MMR vaccine, there is still a risk of a potential outbreak as well as a possible occurrence of congenital rubella syndrome in newborns.

The highest levels of IgG antibodies among seropositive participants were detected in the age group older than 24 years, which belongs to the period of massive epidemics of the rubella disease, indicating that natural acquired immunity results in a higher level of antibodies compared to subjects who have acquired immunity with the vaccine.

It is important to identify susceptible populations, and, in parallel with high coverage of two doses of the MMR vaccine in children, perform additional vaccination of seronegative individuals, especially seronegative women of generative age ("catch-up" campaign) in order to avoid risk of rubella infections in the future.

## Supporting information

S1 Table The incidence of rubella in Vojvodina between 1978 and 2017, and the individual coverage of MMR1 i MMR2 vaccine between 1993 and 2017. (DOC)

S1 Fig Serum level of anti-rubella IgG in different age groups (during the three observed periods) in seropositive participants. (TIF)
