# Supplementary material for: Seroepidemiological study of rubella in Vojvodina, Serbia: 24 years after the introduction of the MMR vaccine in the national immunization programme
Source: PLoS One. 2020 Jan 13;15(1):e0227413. doi: 10.1371/journal.pone.0227413 (PMC6957133; doi:10.1371/journal.pone.0227413)
Supplement: S1 Table — (DOC) [file pone.0227413.s001.doc]

**S1 Table. The incidence of rubella in Vojvodina between 1978 and 2017, and**

**the individual coverage of MMR1 i MMR2** **between 1993 and 2017**

| The endemoepidemic period (preimmunization period) | | | Period of epidemic occurrence of rubella disease and introduction of MMR | | | | Period of sporadic cases of rubella disease | | | |
| --- | --- | --- | --- | --- | --- | --- | --- | --- | --- | --- |
| Years | **Inc. per 100.000 inhabitants** | **Average coverage of MMR1 and MMR2 (%)** |  |  |  |  |  |  |  |  |
| **1978** | **203,5** | **-** |  |  |  |  |  |  |  |  |
| **1979** | **395,8** | **-** | Years | **Inc. per 100.000 inhabitants** | MMR1 | MMR2 |  | | | |
| **1980** | **505,4** | **-** | **1993*** | **74,2** | 97,1% |  | Years | **Inc. per 100.000 inhabitants** | MMR1 | MMR2 |
| **1981** | **447,2** | **-** | **1994** | **653,6** | 92,6% |  | **2006***** | **-** | 96,2% | 97,4% |
| **1982** | **407,5** | **-** | **1995** | **939,1** | 95,5% |  | **2007** | **-** | 97,1% | 90,9% |
| **1983** | **123,4** | **-** | **1996**** | **79,4** | 96,5% | 95,1% | **2008** | **-** | 98,1% | 98,6% |
| **1984** | **442,5** | **-** | **1997** | **32,2** | 97,8% | 98,8% | **2009** | **0,05** | 97,5% | 97,4% |
| **1985** | **1056,9** | **-** | **1998** | **89,8** | 96,4% | 98,4% | **2010** | **-** | 97,4% | 96,8% |
| **1986** | **80,2** | **-** | **1999** | **16,8** | 94,4% | 80,6% | **2011** | **-** | 98,3% | 98,4% |
| **1987** | **57,1** | **-** | **2000** | **12,5** | 82,1% | 60,2% | **2012** | **0,05** | 90,6% | 93,9% |
| **1988** | **87,1** | **-** | **2001** | **13,0** | 97,0% | 95,8% | **2013** | **-** | 96,0% | 85,1% |
| **1989** | **1402,1** | **-** | **2002** | **10,3** | 94,0% | 53,2% | **2014** | **-** | 86,0% | 89,2% |
| **1990** | **40,1** | **-** | **2003** | **3,2** | 96,6% | 89,9% | **2015** | **0,05** | 90,0% | 83,7% |
| **1991** | **52,9** | **-** | **2004** | **1,8** | 97,8% | 98,5% | **2016** | **-** | 89,0% | 90,9% |
| **1992** | **59,8** | **-** | **2005** | **1,1** | 98,1% | 98,8% | **2017** | **-** | 78,0% | 93,2% |

* Introduction of MMR vaccine in the national immunization programme; ** The second dose of the vaccine was initially given at the age of twelve;

*** The second dose was shifted to the age of seven.
